# Fast automated adjoints for spectral PDE solvers

**DOI:** 10.1073/pnas.2530440123

**Published:** 2026-04-10

**Authors:** Calum S. Skene, Keaton J. Burns

**Affiliations:** ^a^Department of Applied Mathematics, University of Leeds, Leeds LS2 9JT, United Kingdom; ^b^School of Physics and Astronomy, The University of Edinburgh, Edinburgh EH9 3FD, United Kingdom; ^c^Department of Mathematics, Massachusetts Institute of Technology, Cambridge, MA 02139; ^d^Center for Computational Astrophysics, Flatiron Institute, New York, NY 10010

**Keywords:** automatic differentiation, PDE-based optimization, spectral methods, computational fluid dynamics, adjoint sensitivity analysis

## Abstract

We present an automated procedure for computing model gradients for partial differential equation (PDE) solvers built on sparse spectral methods, a broad class of numerical techniques widely used in the study of fluid dynamics, continuum mechanics, waves, and pattern formation across disciplines. Our approach applies reverse-mode automatic differentiation to symbolic graph representations of PDEs and efficiently constructs adjoint solvers that retain the speed and flexibility of modern Fourier and polynomial spectral methods. We demonstrate the advantages of this approach with a comprehensive implementation in the open-source Dedalus framework. This work uniquely provides a differentiable spectral solver that supports a broad class of equations, geometries, and boundary conditions, and runs efficiently in parallel. It enables users to compute gradients and perform PDE-based optimization for a wide range of time-dependent and nonlinear models with minimal additional code. We demonstrate this system’s capabilities using canonical problems from the literature, showing both strong performance and practical utility for a wide variety of optimization tasks. By integrating automatic adjoints into a flexible solver, our work enables researchers to perform sensitivity analyses in spectral simulations with ease and efficiency.

## Significance

Scientific computing increasingly requires both accurate simulations of physical systems as well as optimizing them over many parameters for tasks such as inverse modeling, system control, and variational data assimilation. Adjoint methods enable these optimizations by efficiently computing model gradients, but they traditionally require laborious and problem-specific derivations. Here, we present a framework that automatically generates discrete adjoints of partial differential equation (PDE) solvers based on spectral discretizations. Spectral methods are widely used for their speed and accuracy, yet they have lacked a flexible and automated approach for performant adjoint computations. Our framework unlocks fast and scalable sensitivity analysis for spectral simulations, enabling efficient PDE-based optimization across applications including fluid dynamics, plasma physics, biology, and the Earth sciences.

Numerical modeling has become a ubiquitous tool across every discipline of science and engineering. However, systematically using forward models to enhance scientific understanding often requires costly optimizations over model inputs and parameters. Adjoint state methods provide an efficient and systematic way to compute the model gradients needed for such model-based optimization. Thus, the ability to compute adjoints forms the foundation of many scientific studies that would otherwise be infeasible. Indeed, the recent rise of machine learning and artificial intelligence as a transformative technology would not have been possible without adjoint methods, which form the basis of the backpropagation algorithms needed to train models. Beyond machine learning, adjoint methods have a rich history in scientific computing. They are an indispensable tool in nonmodal stability theory (1, 2), parametric sensitivity analysis (3, 4), and weather forecasting via four-dimensional data assimilation (4D-Var) (5, 6). Adjoint techniques have also been applied in countless optimization settings—for example, to design aerodynamic structures (7), mitigate thermoacoustic instabilities (8), optimize stellarator designs for nuclear fusion reactors (9, 10), and solve inverse problems in geophysics (11), among many other applications.

Despite their evident utility, adjoint-based computations remain underutilized in many fields, largely due to the difficulty of computing the adjoint operator, which is often a tedious and error-prone process. For numerical solvers of partial differential equations (PDEs) (e.g., fluid flow or electromagnetics), there are two main strategies for obtaining adjoints. One approach is to analytically derive an adjoint system by integrating the governing equations by parts in both space and time; the resulting continuous adjoint equations are then discretized and solved numerically. Alternatively, the PDE can be discretized first, and then the adjoint of the resulting discrete system can be computed using tools like automatic differentiation (AD). These are known as the continuous and discrete adjoint approaches, respectively, each with their own advantages and disadvantages (see refs. 12 and 13 for detailed discussions).

Several open-source simulation codes now include built-in adjoint functionality (often termed “differentiable” codes), using a mix of continuous and discrete approaches. This includes SU^2^ (14), FEniCS (15), Firedrake (16), $\Phi_{\text{Flow}}$ (17), simsopt (10), OpenFOAM (18), Exponax (19), JaxFluids (20, 21), JAX-CFD (22, 23), Trixi.jl (24), and Julia’s SciML ecosystem (25). The majority of these PDE solvers focus on specific equation sets and are tied to particular applications or geometries. Notably, FEniCS and Firedrake are different in that they are high-level finite-element libraries designed for a wide variety of models. To use these packages, the governing equations must be specified in variational (weak) form via the “unified form language” (26), which are then discretized by the library. Adjoint computations are executed automatically using the dolfin-adjoint framework (27, 28). This approach has enabled numerous adjoint-based studies across a range of PDE applications (e.g., refs. 29 and 30).

While finite-element methods excel for complex geometries, global spectral methods are a popular and complementary alternative for problems in simpler geometries, such as turbulent fluid flow in a box or a sphere. These methods offer exponential accuracy as resolution is increased, and they facilitate efficient elliptic solves for differential-algebraic equations as well as implicit time-stepping for stiff problems (31). Spectral methods are widely used in fundamental research and underpin the world’s largest turbulence simulations (32) and state-of-the-art weather forecasting models (33). The JAX-CFD code includes support for automatic differentiation of Fourier spectral solvers for incompressible hydrodynamics, but does not support high-order timestepping, nonperiodic boundary conditions, steady-state simulations, or distributed-memory parallelism. Beyond classical Fourier methods, recent developments in sparse polynomial spectral methods have extended Fourier-like speed and accuracy to simple geometries in Cartesian (34), polar (35), and spherical coordinates (36). These advances have enabled cutting-edge simulations of biophysical, geophysical, and astrophysical flows (e.g., refs. 37, 38, 39).

Here, we present a numerical approach to automatically generate discrete adjoints for the entire family of fast global spectral methods, implemented in the open-source Dedalus framework (40). Like FEniCS and Firedrake, Dedalus is a high-level library that discretizes and solves user-defined PDEs. However, Dedalus allows users to specify systems of equations in strong form via a simple interface that is both flexible and accessible to computational scientists across many disciplines. Dedalus includes many spectral bases and has solver paths for eigenvalue problems (EVPs), linear boundary value problems (LBVPs), nonlinear boundary value problems (NLBVPs), and initial value problems (IVPs). Dedalus is written in Python, with compiled extensions for performance-critical routines, and it automatically handles distributed-memory parallelism via MPI. These features allow Dedalus simulations to be easily prototyped on a laptop and then run at scale on high-performance computing clusters.

Our procedure applies AD to the high-level equation representation in Dedalus and fuses discrete adjoint routines for the underlying solver components. By exploiting the modularity of the codebase, we have made Dedalus differentiable without needing to rewrite the library to be compatible with any particular AD toolchain. However, our implementation can still easily interface with external optimization libraries [e.g., Manopt (41)] and integrators [e.g., PETSc (42)], and allows combining sparse spectral solvers with machine learning frameworks [e.g., PyTorch (43)], which inherently require differentiable simulators for training.

In this article, we demonstrate the capabilities of our adjoint framework by using Dedalus to solve several representative optimization problems drawn from a variety of scientific domains. These examples highlight the efficiency, flexibility, and ease of use of our differentiable extension of Dedalus. By working with discrete adjoints, our method automatically handles any boundary conditions, constraints, and gauge choices, eliminating the difficulties these pose for continuous-adjoint approaches. Importantly, in all these examples, the gradient computation requires minimal user intervention: Only a few additional lines of code are needed to obtain the gradient of any cost functional evaluated on a Dedalus solution. Overall, our work makes adjoint-based optimization readily accessible to scientists using spectral methods across many workflows and applications.

## Spectral Adjoints

### Continuous vs. Discrete.

The fundamental definition of an adjoint is mathematically simple: An adjoint operator $A^{†}$ to a linear operator A is one that satisfies $\left\langle x,Ay\right\rangle =\left\langle A^{†}x,y\right\rangle$ for all x and y, where ⟨·,·⟩ is a suitably chosen inner product. Efficiently solving the adjoint of a numerical model’s Jacobian yields the model’s gradients. Continuous methods discretize the infinite dimensional adjoint, while discrete methods discretize the forward operator and then take its finite-dimensional adjoint.

The continuous adjoint has the advantage of producing the true adjoint of the governing equations. However, it generally does not provide the exact gradient of the discrete forward model (even if the forward model is well resolved), and deriving it—either manually or automatically—is difficult for constrained systems or complex boundary conditions. Implementing a continuous adjoint also requires writing a separate solver in addition to the original model. The discrete adjoint, on the other hand, yields the correct gradient for the discrete forward model. This advantage often translates into better convergence in outer-loop optimizations, although it can introduce instabilities when the forward model is underresolved (12, 25).

Several studies have manually implemented continuous adjoints for specific PDEs in Dedalus using its symbolic equation-entry interface. For example, adjoint methods have been used to explore the similarities between minimal seeds and instantons (44), to solve inverse problems (45), and to identify subcritical geodynamo solutions (46). In a recent study (47), continuous and discrete adjoints were compared across several Cartesian problems in Dedalus, highlighting the advantages of the discrete approach—especially when used with optimization methods that benefit from the discrete adjoint’s superior convergence. In each of these cases, implementing the adjoint solver demanded significant user effort (analytical derivations or manual coding of the discrete adjoint), particularly for bounded domains and high-order time-stepping schemes.

Importantly, models implemented within AD frameworks [e.g., Enzyme (48), JAX (49), or Tapenade (50)] can automatically construct discrete adjoints directly from forward-model code, contributing to their growing popularity in recent years. However, AD toolchains often face limitations in programming-language interoperability and hardware support. Moreover, high-performance library routines (e.g., FFTs and linear algebra kernels) typically require user-defined custom derivatives or “chain rules,” since explicitly providing Jacobian actions is often more efficient and numerically stable than tracing the full forward computation. For the highly structured operations common in high-order PDE solvers, such custom rules may be necessary for most of the computational pipeline, diminishing the benefits of end-to-end AD.

Here, we take the approach of leveraging the existing flexible, high-level code structures that enable Dedalus to forward model wide varieties of PDEs. We define adjoints of the various solver types at the outer level, and use the code’s internal computational graphs to perform a highly structured form of reverse-mode AD that combines the discrete adjoints of the low-level operator implementations. Our approach eliminates the barriers associated with manually deriving continuous adjoint systems and produces discrete adjoints without requiring modifications of the forward solver or relying on external AD libraries. This combination enables Dedalus to automatically compute the discrete adjoint for every available solver type, geometry, and time-stepping scheme in an efficient and platform-independent manner.

### Sparse Spectral Methods.

Spectral methods form finite-dimensional discretizations of PDEs by expanding the unknown solutions in a trial basis and projecting the equations against a test basis, enforcing the strong-form PDE via a weighted-residual method. Recent advances in the field have established optimal choices for the test and trial bases to produce maximally sparse discretizations for wide ranges of equations and domains (34–36, 51–53). Linear operators can be directly discretized into sparse (often narrowly banded) matrices, which are fast to apply or invert. Nonlinear terms can be efficiently evaluated on a collocation grid using fast transforms (e.g., FFTs). Boundary conditions and other constraints can be applied by modifying the discretized systems, or at the continuous level through the use of Lagrange multipliers and tau terms (54).

Solvers for wide classes of BVPs and IVPs can then be readily automated as series of sparse linear system solves against explicitly computed right-hand sides (RHSs); for instance, this encapsulates both Newton iterations for BVPs and mixed implicit-explicit (IMEX) timestepping for IVPs. For notational brevity, we will denote such a forward solution as a vector of solution coefficients X satisfying the equation F(X,p)=0, where p is a vector of problem parameters. In *SI Appendix*, we provide more details on how EVPs and IVPs are handled, but we will illustrate the process using this simple notation that more naturally matches the form of BVPs. The goal of the discrete adjoint program is to efficiently compute gradients of solution properties with respect to the parameters, and to do so by reusing the flexible and efficient computational motifs (sparse direct solvers and fast transforms) that enable the automated forward solution of PDEs with spectral methods.

### Efficiently Calculating Gradients.

Here, we summarize how discrete adjoints are used to obtain the derivative with respect to parameters p—which could be forcing terms, initial conditions, or equation parameters—of a functional applied to the spectral solution of a PDE. We denote the target functional as $J(\tilde{X},p)$, where $\tilde{X}$ is a generic vector in the finite-dimensional solution space. We seek to compute the derivative at a point $\tilde{\text{X}}=X(p)$ which solves the discretized PDE with the specified parameters, i.e., F(X(p),p)=0. As a functional purely of the parameters, we define J(p)=J(X(p),p). Directly differentiating this functional by the chain rule yields

[1] $$\begin{array}{c} \frac{\text{d}J}{\text{d}p}=\frac{\partial J}{\partial p}+\frac{\partial J}{\partial \tilde{X}}\cdot \frac{\text{d}X}{\text{d}p}, \end{array}$$

where the partial derivatives are evaluated at (X(p),p). The difficulty in computing this gradient explicitly is the last term, dX/dp, since directly calculating this Jacobian by finite differences requires solving the forward equation for every element of p, which becomes prohibitively expensive as the number of parameters increases.

To obtain the gradient with a tractable computational procedure, the adjoint state method approaches this as a constrained optimization problem (for a detailed review, see refs. 11 and 55). The associated Lagrangian is

[2] $$\begin{array}{c} L(\tilde{X},\tilde{Y},p)=J(\tilde{X},p)-\langle \tilde{Y},F(\tilde{X},p)\rangle , \end{array}$$

where $\tilde{X}$ and $\tilde{Y}$ are generic vectors. Applying the chain rule directly to L gives

[3] $$\begin{array}{c} \frac{\text{d}L}{\text{d}p}=\frac{\partial L}{\partial p}+\frac{\partial L}{\partial \tilde{X}}\cdot \frac{\partial \tilde{X}}{\partial p}+\frac{\partial L}{\partial \tilde{Y}}\cdot \frac{\partial \tilde{Y}}{\partial p}. \end{array}$$

By construction, the last term in this expression will be zero when evaluating the gradient at $\tilde{\text{X}}=X\left(p\right)$ because $\left(\partial L/\partial \tilde{\text{Y}}\right){|}_{X}=-F\left(X,p\right)=0$. Calculating $\partial L/\partial \tilde{\text{X}}$ yields

[4] $$\begin{array}{c} \frac{\partial L}{\partial \tilde{X}}=\frac{\partial J}{\partial \tilde{X}}-{\left(\frac{\partial F}{\partial \tilde{X}}\right)}^{†}\tilde{Y}, \end{array}$$

where † indicates the Hermitian adjoint. This term can be set to zero by choosing $\tilde{Y}=Y(p)$, where Y(p) satisfies the adjoint state equation,

[5] $$\begin{array}{c} {\left(\frac{\partial F}{\partial \tilde{X}}\right)}^{†}Y(p)=\frac{\partial J}{\partial \tilde{X}}, \end{array}$$

where again all partials are evaluated at (X(p),p). Hence, by setting $\tilde{X}=X(p)$, $\tilde{Y}=Y(p)$, and by using the fact that L(X(p),·,p)=J(p), we obtain

[6] $$\begin{array}{c} \frac{dJ}{dp}=\frac{dL}{dp}=\frac{\partial L}{\partial p}=\frac{\partial J}{\partial p}-\langle Y,\frac{\partial F}{\partial p}\rangle . \end{array}$$

Eq. **6** is key to the adjoint method and shows that by first solving for Y(p) we can efficiently obtain the gradient of J, since the dimension of the adjoint state is independent of the number of parameters.

To summarize, in the adjoint state method:

1. The forward problem is first solved to determine X(p).
2. The adjoint problem (Eq. **5**) is then solved for Y(p).
3. Finally, the right-hand side of Eq. **6** is computed to determine the gradient dJ/dp.

The first (forward) step is already automated in Dedalus using optimally sparse spectral methods. We have now automated the second and third (adjoint) steps, enabling automatic PDE-based optimization through a simple user interface. The principal technical requirements are

- Implementing fast direct solvers for the Jacobian adjoints appearing on the left-hand side of Eq. **5**. This is achieved by reusing sparse factorizations from the forward solver stages.
- Adding fast evaluations of the derivative terms on the right-hand side of Eqs. **5** and **6**. This is achieved by implementing matrix-free vector-Jacobian products (VJPs) for arbitrary nonlinear operator graphs, via a custom high-level AD system that leverages fast discrete adjoints for the individual operator components.

**Fig. 1.**
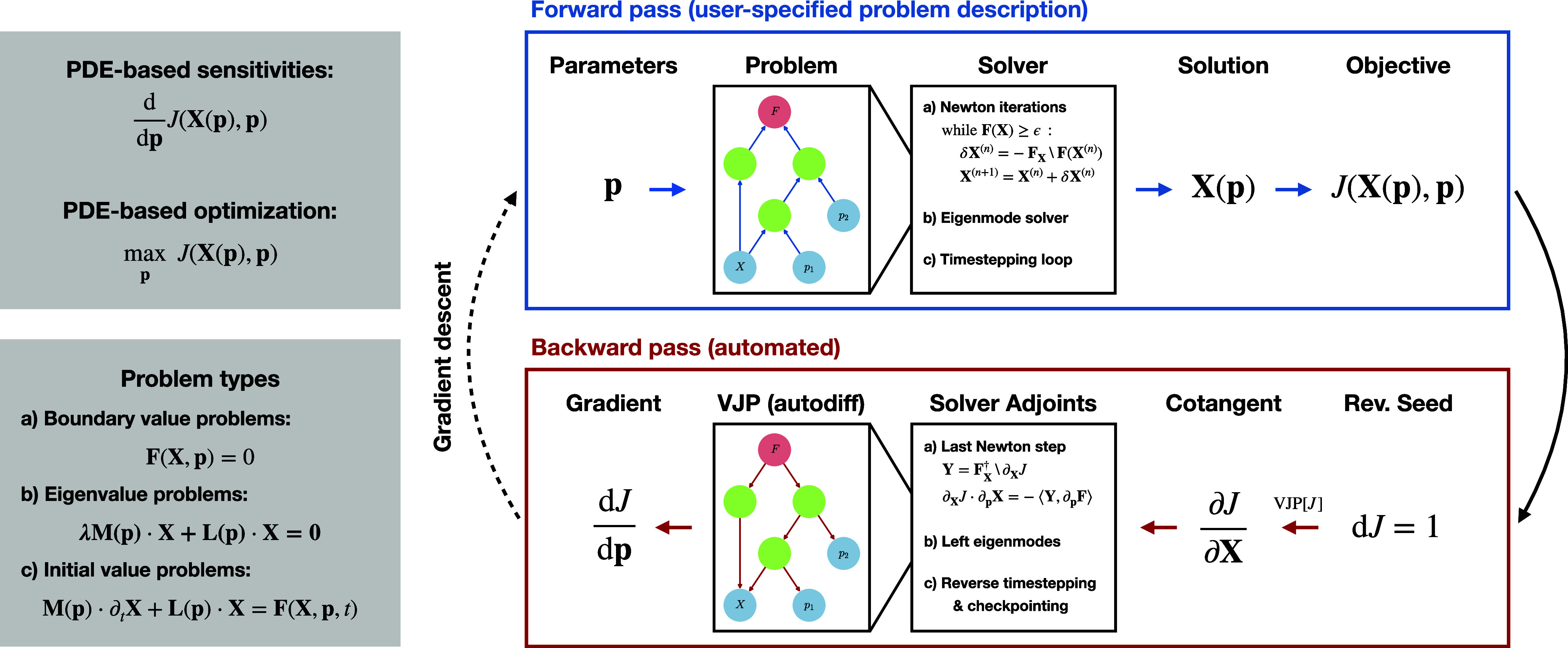
Depiction of the adjoint-looping process to perform efficient PDE-based optimization using automatic discrete adjoints in Dedalus. *Left*: We seek to optimize a functional J of the solution X of a PDE (BVP, EVP, or IVP) over parameters p. *Top Right*: Dedalus already enables solving general PDEs with fast spectral methods with operator graphs representing the PDE (forward pass). *Bottom Right*: We have now automated the adjoint (backward pass) by reversing the high-level solver control flow and implementing efficient vector-Jacobian products (VJPs) on the operator graphs, akin to reverse-mode automatic differentiation. The result is an optimally fast computation of the discrete model gradient.

The workflow of the adjoint looping process using these tools is illustrated in Fig. 1. More details about the technical implementations are provided in *Materials and Methods* and *SI Appendix*.

## Results

To demonstrate the simplicity and flexibility of our approach to computing discrete adjoints for sparse spectral solvers, we have created fast implementations using Dedalus of several canonical problems in PDE-based optimization. We have chosen examples using a variety of solver types, illustrating different scenarios in which gradient information is utilized. They are also chosen to cover a range of application areas and domain geometries, showcasing the flexibility of our framework and highlighting its suitability for enhancing a wide range of PDE-based modeling studies. The scripts for these examples, among others, are available on GitHub (*Data, Materials, and Software Availability*).

### Parametric Sensitivity and Numerical Continuation.

Since adjoints provide efficient access to gradient information, they are an ideal tool for parametric sensitivity analyses where we seek the direct impact of problem parameters on the PDE solution. Here, we consider the process of computing the neutral stability curve for plane Poiseuille flow (56, 57), a canonical fluid dynamics problem that describes pressure-driven flow between two flat plates situated at y=±1. In nondimensional variables, the stability problem can be written as an eigenvalue problem involving the velocity and pressure perturbations $u^{\prime }$ and $p^{\prime }$ as

[7] $$\begin{array}{cc} & \lambda u^{\prime }+u_{0}\cdot \nabla u^{\prime }+u^{\prime }\cdot \nabla u_{0}=-\nabla p^{\prime }+\frac{1}{\text{Re}}\nabla^{2}u^{\prime },\\ & \nabla \cdot u^{\prime }=0,\\ & u^{\prime }\left(y=\pm 1\right)=0, \end{array}$$

where $u_{0}=\left(1-y^{2}\right){\hat{e}}_{x}$ is the base flow and Re is the Reynolds number. The real part of the eigenvalue λ is the growth rate (γ=Rλ), and the imaginary part gives the oscillation frequency. As the base flow is independent of x, this stability problem is separable over streamwise wavenumbers α. For what follows, we consider only 2D perturbations (with no spanwise z-dependence), and seek to find the sets of parameters (α, Re) such that the maximum growth rate is zero, indicating neutral stability.

To accelerate the process of computing the neutral curve, we utilize discrete model adjoints in Dedalus in two ways. First, we guess a point close to the neutral curve and use the EVP solver and its adjoint to compute the largest growth rate γmax(Re,α) and its parametric gradient at this point. Using a Newton solver, we can then efficiently find a point on the neutral curve where $\gamma_{\max}=0$. Second, to construct a guess for a new point on the neutral curve, we use the parametric gradient to find the parametric tangent to the neutral curve, from which a new guess can be estimated. By repeating this process, we can efficiently trace the neutral curve without difficulties continuing around turning points.

**Fig. 2.**
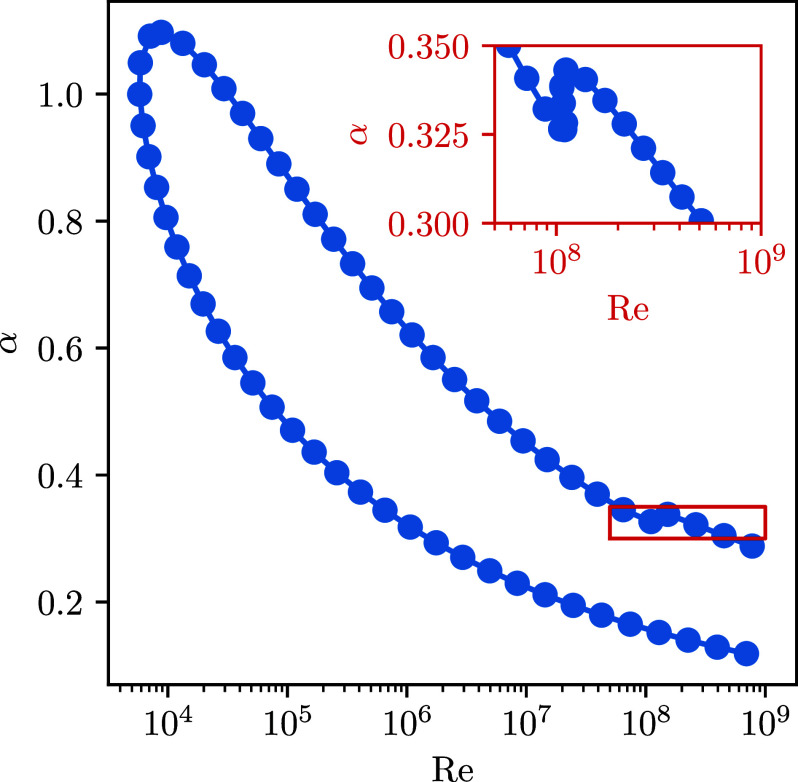
Neutral stability curve for plane Poiseuille flow obtained with adjoint-based eigenvalue sensitivities and numerical continuation. Each point on the curve has a growth rate of zero to machine precision. The parameters are the horizontal wavenumber α and the base-flow Reynolds number Re.

Fig. 2 shows the result of this procedure, reproducing the classical stability boundary. Each point indicated on this curve has a growth rate of zero to machine precision and was calculated with approximately five eigenvalue solves. This leads to a very efficient calculation of the stability boundary without requiring a multidimensional grid search. While this equation only contains two parameters, the adjoint method allows quick calculations of eigenvalue sensitivities with respect to potentially many more. Furthermore, the adjoint implementation reuses the LU decomposition from the forward problem, providing significant computational savings over finite differences, which would require new forward solves and factorizations.

### Nonlinear Optimization.

Propagating sensitivities through nonlinear evolutionary equations is possible with adjoint looping, where the backward pass corresponds to a reverse-time integration using the time-dependent linearization of the forward model. This technique allows optimizing, e.g., final-time functionals with respect to equation parameters and initial conditions. To demonstrate the capabilities of automatic differentiation in Dedalus as a tool for nonlinear optimization, we consider the problem of optimizing dynamo action in a ball with insulating boundary conditions. We follow Chen et al. (58), which utilizes the optimization procedure established in ref. 59 for Cartesian domains. This is an important and challenging example of using nonlinear optimization to assess the stability of fluid flows [see the review (60) and references therein].

In the astrophysical context, dynamo theory deals with the growth of magnetic fields via the motion of a conducting fluid—an important process in planets, stars, accretion disks, and galaxies (for a review, see ref. 61). This problem is governed by the induction equation,

[8] $$\begin{array}{cc} & \frac{\partial B}{\partial t}-\eta \nabla^{2}B=\nabla \times \left(u\times B\right),\\ & \nabla \cdot B=0, \end{array}$$

where B(t) is the magnetic field, u is the fluid velocity, and η is the magnetic diffusivity. Although this is a linear evolution equation for B(t) when the velocity field is fixed, it is nonlinear as a joint optimization over u and B(0), and obtaining growing solutions can be a highly nontrivial and subtle task.

Scaling length with the domain radius R, velocity with ΩR for a prescribed vorticity scale Ω, time with $R^{2}/\eta$, and magnetic field with an arbitrary strength B, the induction equation includes a single nondimensional parameter, the vorticity-based magnetic Reynolds number $\text{Rm}\equiv R^{2}\Omega /\eta$. In order to ensure the magnetic field remains solenoidal, we formulate the problem using a magnetic vector potential A(t), where B=∇×A, as

[9] $$\begin{array}{cc} & \frac{\partial A}{\partial t}-\nabla^{2}A+\nabla \psi =\;\text{Rm}\;u\times B,\\ & \nabla \cdot A=0. \end{array}$$

These equations include a scalar field ψ that is fixed by the Coulomb gauge condition. Since the temporal eigenmodes of B correspond to temporal eigenmodes of A with the same growth rate, optimizing the growth of A beyond initial transients will yield the same optimal velocity field u as directly optimizing the growth of B.

Defining ${\left\|X\right\|}^{2}=\left(1/V\right)\int X\cdot X\;\;\text{d}V$ and following ref. 58, we formulate the optimization problem as maximizing

[10] $$\begin{array}{c} J={\log\|A\left(T\right)\|}^{2} \end{array}$$

with constraints ‖A(0)‖=1 and ‖ω‖=1, where ω=∇×u is the vorticity field. Note that the norm of the vorticity, rather than the velocity, is fixed due to the results of ref. 62. We specify vacuum boundary conditions for the magnetic field, which can be written in terms of the spherical harmonic decomposition of A as

[11] $$\begin{array}{c} \frac{\partial A_{\ell ,\sigma }}{\partial r}+\frac{\ell +1+\sigma }{r}A_{\ell ,\sigma }=0\;\;\text{at}\;r=1, \end{array}$$

where ℓ is the spherical harmonic degree and we have used the regularity component index σ∈{−1,1,0} (36). Since the norm of ω is constrained, we obtain u from ω by solving the auxiliary LBVP

[12] $$\begin{array}{cc} & \nabla \times (\nabla \times u)+\nabla \chi =\nabla \times \omega ,\\ & \nabla \cdot u=0, \end{array}$$

together with no-slip conditions on u. This ensures that the velocity field remains divergence-free and satisfies appropriate boundary conditions.

We utilize the Pymanopt (63) library to perform the optimization on the product manifold $\left(A\left(0\right),\omega \right)\in \Pi =V_{W}\times V_{W}$, where $V_{W}=\left\{x\;|\;x^{T}Wx=1\right\}$ is a generalized Stiefel manifold with a weight matrix W corresponding to the discretized $L^{2}$ norm. Through this formulation, the internal Pymanopt solver uses a Riemannian conjugate gradient method with line search (64), resulting in rapid convergence and simple enforcement of the norm constraints.

**Fig. 3.**
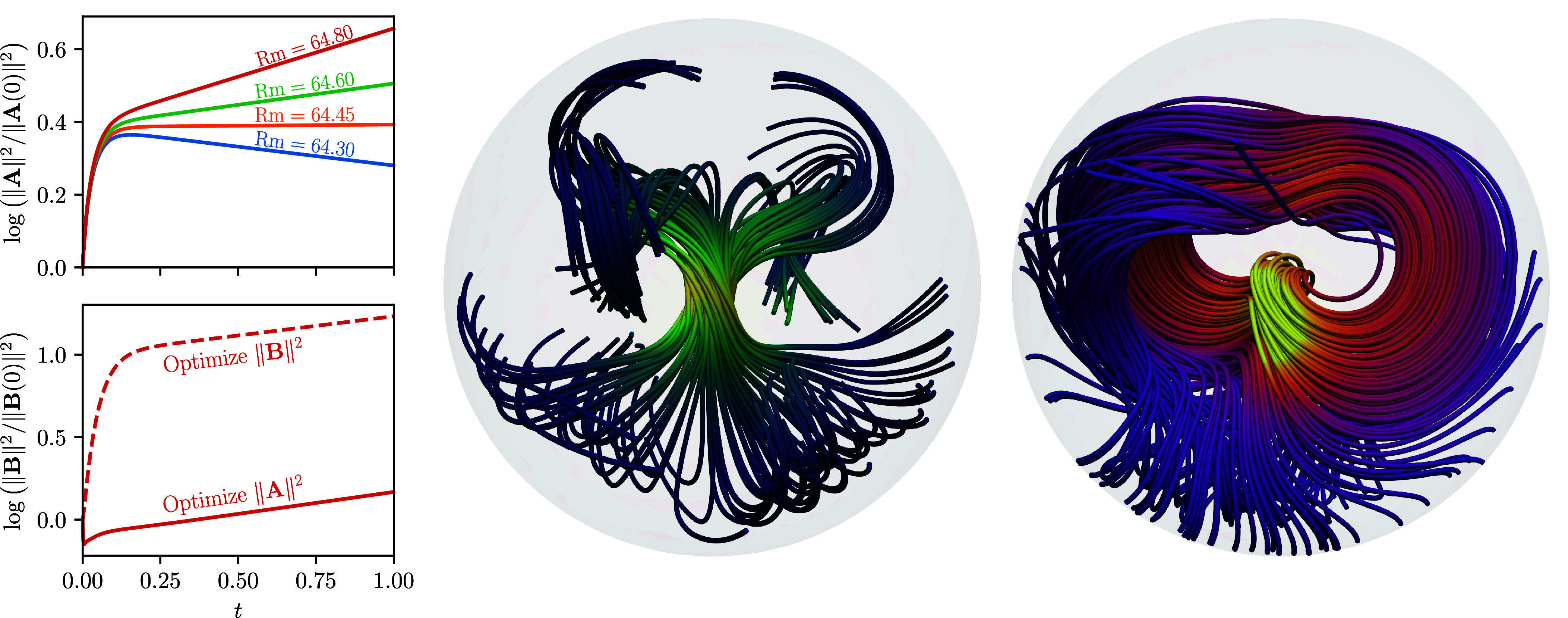
Optimal kinematic dynamo solutions in the ball from nonlinear optimization via adjoint looping, following Chen et al. (65). *Top Left*: Time series showing the result of optimizing the vector potential norm at different magnetic Reynolds numbers. *Bottom Left*: Time series comparing optimization of the magnetic field norm vs. the vector potential norm. *Middle*: Streamlines of the optimal velocity field for Rm=64.45. *Right*: Field lines of the optimal magnetic field at t=1 for Rm=64.45.

The results of the optimization procedure are shown in Fig. 3. From the time series, we see that for all magnetic Reynolds numbers there is a short period of transient growth (up to t≈0.1), followed by an exponential period in which only the mode with the largest growth rate remains. For Rm≲64.45, the flow is stable and this growth rate is negative, whereas for Rm≳64.45 the growth rate is positive. For Rm≈64.45 the growth rate is approximately zero, indicating that this is the critical magnetic Reynolds number. The figure also shows the streamlines and field lines of the optimal velocity and magnetic fields, respectively. We see that the flow is mainly concentrated near the center of the ball, where a large increase in flow velocity occurs with a twisting motion. These results are all in close agreement with ref. 58.

To highlight the flexibility of our method, we also perform the optimization problem of maximizing

[13] $$\begin{array}{c} J={\log\|B\left(T\right)\|}^{2} \end{array}$$

subject to ${\left\|B\left(0\right)\right\|}_{2}=1$. This is done by solving the induction equation directly for B. Fig. 3 shows the resulting magnetic energy under this optimization compared to the initial optimization using A. As expected, both energies show the same growth rates after the initial transient. However, the amount of transient growth is notably different, with the magnetic-energy-based optimization leading to substantially more absolute growth. This example emphasizes the flexibility of our automated adjoint capabilities for spectral solvers, which allow users to easily examine problems from different viewpoints using different equation formulations.

### Resolvent Analysis.

Resolvent analysis is a powerful modal analysis technique (66), whose applications to turbulence were pioneered by McKeon and Sharma (67). In their setup, the flow field is decomposed into a stationary mean flow $\overset{¯}{u}$ and fluctuations $u^{\prime }$, such that $u=\overset{¯}{u}+u^{\prime }$. Substituting this into the Navier–Stokes equations and Fourier transforming in time results in the following coupled systems of equations:

[14] $$\begin{array}{cc} & i\omega {\hat{u}}^{\prime }+\overset{¯}{u}\cdot \nabla {\hat{u}}^{\prime }+{\hat{u}}^{\prime }\cdot \nabla \overset{¯}{u}+\nabla \hat{p}-\frac{1}{\text{Re}}\nabla^{2}{\hat{u}}^{\prime }=F\left[u^{\prime }\cdot \nabla u^{\prime }\right]\left(\omega \right),\\ & \nabla \cdot {\hat{u}}^{\prime }=0, \end{array}$$

and

[15] $$\begin{array}{cc} & \overset{¯}{u}\cdot \nabla \overset{¯}{u}+\nabla \overset{¯}{p}-\frac{1}{\text{Re}}\nabla^{2}\overset{¯}{u}=F\left[u^{\prime }\cdot \nabla u^{\prime }\right]\left(0\right),\\ & \nabla \cdot \overset{¯}{u}=0, \end{array}$$

where we denote the Fourier transform at frequency ω as F[·](ω). Eq. **14** shows that fluctuations at frequency ω are driven by triadic interactions, represented by the right-hand side. In turn, Eq. **15** shows that the zero-frequency component of these triadic interactions sustains the mean flow.

Instead of solving the fully coupled system, mean flow data can be obtained from simulations or experiments. In doing so, Eq. **14** can be reduced to the form

[16] $$\begin{array}{c} \left(i\omega M+L\right){\hat{u}}^{\prime }=\hat{f}, \end{array}$$

where M is a mass matrix, L is a linear operator, and the triadic interactions are written as a forcing term $\hat{f}$.

Resolvent analysis treats $\hat{f}$ as a generic forcing term arising from turbulence and examines Eq. **16** as an input–output system in which a transfer function $H={\left(i\omega M+L\right)}^{-1}$ maps a forcing $\hat{f}$ to its driven response ${\hat{u}}^{\prime }$. This transfer function H is known as the resolvent. Of interest are the leading singular values and vectors of this operator, i.e., $\left(\sigma_{i},{\hat{u}}_{i}^{\prime },{\hat{f}}_{i}\right)$ such that $H{\hat{f}}_{i}=\sigma_{i}{\hat{u}}_{i}^{\prime }$ and the vectors ${\hat{u}}_{i}^{\prime }$ and ${\hat{f}}_{i}$ are orthonormal under the $L^{2}$ (energy) norm. The gains $\sigma_{i}$ measure the amount of amplification a particular forcing induces in the flow response. In situations where the largest gain $\sigma_{1}$ is much greater than the next largest $\sigma_{2}$, the flow is deemed low-rank, and a generic forcing $\hat{f}$ is likely to produce a response that resembles ${\hat{u}}_{1}^{\prime }$ due to the orthogonality of the singular vectors.

Hence, a resolvent analysis reveals two key insights. First, it systematically identifies the dominant frequencies of turbulent fluctuations, where the gains are maximized. Second, for frequencies at which the flow is found to be low-rank, the leading response mode is expected to resemble coherent structures found in the turbulent flow. Furthermore, by examining the corresponding forcing mode, one can gain important information relevant for flow control (see, e.g., refs. 68 and 69). While we focus here on resolvent analysis for turbulent flows (about the mean flow), it is worth noting that resolvent analysis for steady flows (about fixed points) is also an important concept in nonmodal stability analysis (1, 70), predating its use in turbulence, and serves as the driven counterpart to transient growth analysis.

Using our automatic adjoint routines in Dedalus, we reproduce the resolvent analysis for turbulent pipe flow following ref. 67. Since the mean flow is obtained by averaging over ϕ and z, it depends only on the radial coordinate, allowing us to solve for the forcing and response independently for each azimuthal wavenumber m and streamwise wavenumber k. We use Dedalus to compute the action of both H and $H^{†}$ for given m and k in a cylindrical geometry. This is done using a linear boundary value problem (LBVP) to apply H via solving the associated matrix pencil, and applying the adjoint via the vector-Jacobian product of the LBVP. Together, these routines are used to compute the leading singular vectors via the SciPy sparse SVD. The mean flow is taken from experimental data at Re=74,345 (71).

**Fig. 4.**
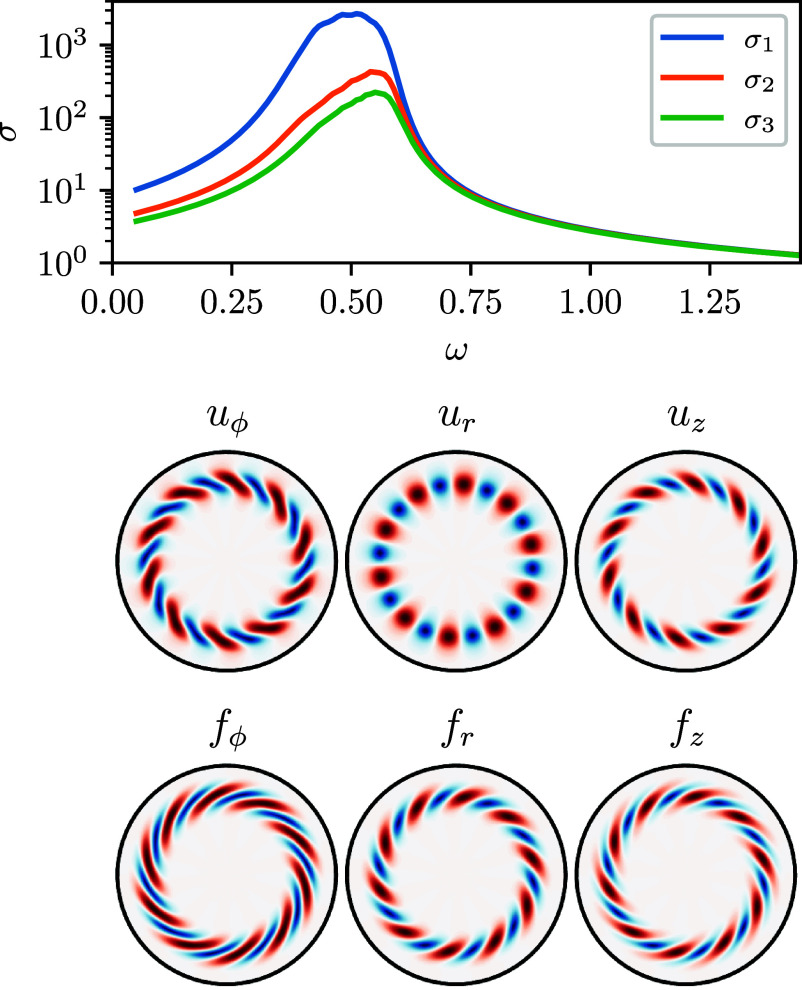
Resolvent analysis for turbulent pipe flow at Re=74,345, following McKeon and Sharma (67), for axial wavenumber k=1 and azimuthal wavenumber m=10. *Top*: Leading gains (singular values) as a function of frequency, showing the optimal response at ω≈0.5. *Bottom*: Optimal forcing and response modes, showing structures similar to those observed in experimental fluctuations.

Fig. 4 shows the resolvent results for azimuthal wavenumber m=10 and streamwise wavenumber k=1, in excellent agreement with ref. 67. The figure illustrates that over a wide range of frequencies, the flow is low-rank, with an order-of-magnitude separation between $\sigma_{1}$ and $\sigma_{2}$. The optimal response near the peak gain at ω=0.5 is concentrated near the wall and resembles large-scale motions observed in turbulent pipe experiments. This example demonstrates the ease of setting up and performing a resolvent analysis using a differentiable spectral code. Since many turbulent experimental configurations—such as pipe flow, channel flow, and Taylor–Couette flow—can be modeled spectrally, there is a clear advantage to complementing these experiments with resolvent analysis. In particular, we envision that resolvent analyses for fluids not modeled by the incompressible Navier–Stokes equations (e.g., compressible, conducting, non-Newtonian, or viscoelastic fluids) will be made more accessible by these developments, which significantly ease the burden of implementation.

### Phase Reduction Analysis.

Phase-reduction analysis is a technique with origins in mathematical biology and neuroscience that is used to study phase dynamics and synchronization in oscillating systems (72, 73). More recently, it has gained traction in the fluid mechanics community (74), where it has been applied to assess the synchronization properties of fluid–structure interactions (75), thermoacoustic instability (76), and to design flow actuation strategies in aeronautical engineering applications (77, 78).

For a stable limit-cycle solution with period T, the notion of phase can be defined as follows. The phase θ is first defined for states on the limit cycle as 2πt/T for t∈[0,T]. In other words, for a state on the limit cycle, the phase variable θ∈[0,2π] assigns a scalar label to each state, and satisfies the simple ODE $\dot{\theta }=2\pi /T$ on the cycle. This definition can be extended to states in the vicinity of the limit cycle by using the fact that the cycle is stable, and therefore any small perturbation returns to it. Hence, for a state near the limit cycle, we define its phase as the phase of the point on the limit cycle whose trajectory it matches asymptotically as t→∞. Under this definition, the phase dynamics near the limit cycle are governed by the ODE

[17] $$\begin{array}{c} \dot{\theta }=\frac{2\pi }{T}+z\left(\theta \right)\cdot h\left(\theta \right), \end{array}$$

where h(θ) represents a small perturbation to the system and z(θ) is the phase sensitivity function, whose computation is the central task in a phase-reduction analysis. By obtaining z, the phase response can be determined independently of the specific form of the perturbation, making this a powerful technique for analyzing the phase and synchronization dynamics of oscillators.

As an example of how adjoints enable spectral computations for phase-reduction analysis, we consider the FitzHugh-Nagumo model (79, 80):

[18] $$\begin{array}{cc} & \frac{du}{dt}=u-\frac{u^{3}}{3}-v+I,\\ & \frac{dv}{dt}=\epsilon \left(u+a-bv\right), \end{array}$$

a simplified version of the Hodgkin–Huxley model (81). These equations model the firing dynamics of a neuron, with membrane potential u and recovery variable v. The parameters include ϵ (which sets the timescale separation between u and v), the input current I, and constants a and b describing the activation dynamics. For I≠0, the system exhibits slow-fast repetitive firing for a range of parameter values, yielding a stable limit-cycle solution.

Of interest is the phase response of the dynamics about this limit cycle. This includes how perturbations can delay or advance the phase of the solution, synchronization of neurons to external stimuli (82), collective synchronization of coupled neurons (83), and synchronization of oscillators to common white noise (84). The key to understanding all of these phenomena lies in computing the phase sensitivity function, which can be efficiently obtained via adjoint methods (see ref. 73, for example). This connection between the adjoint problem and the phase sensitivity function is essential for efficiently conducting a phase-reduction analysis, particularly in PDE systems, as recently demonstrated for fluid flows (85).

To compute the phase sensitivity function using Dedalus, we first compute the limit-cycle solution $\left(u_{0},v_{0}\right)$. To do this, we discretize Eq. **18** in time using a Fourier basis, and solve it as a nonlinear boundary value problem (NLBVP) to obtain a spectrally accurate periodic solution. Floquet theory then gives that perturbations to this limit cycle $\left(u^{\prime },v^{\prime }\right)$ satisfy the eigenvalue problem

[19] $$\begin{array}{cc} & \frac{du^{\prime }}{dt}+\lambda u^{\prime }=u^{\prime }-u_{0}^{2}u^{\prime }-v^{\prime },\\ & \frac{dv^{\prime }}{dt}+\lambda v^{\prime }=\epsilon \left(u^{\prime }-bv^{\prime }\right), \end{array}$$

where λ is the eigenvalue and where $u^{\prime }$ and $v^{\prime }$ are again discretized using Fourier series in t [the Floquet–Fourier–Hill method (86)]. Since the system is autonomous, it has a neutral eigenvalue λ=0 with corresponding eigenvector $\left({\dot{u}}_{0},{\dot{v}}_{0}\right)$ representing phase shifts along the limit cycle. The phase sensitivity function, which allows the projection of dynamics onto this phase shift, is obtained as the corresponding adjoint eigenvector, normalized such that $\langle z,{\left({\dot{u}}_{0},{\dot{v}}_{0}\right)}^{T}\rangle =1$. Thus, the phase sensitivity function can be computed in Dedalus simply by solving the adjoint of Eq. **19**.

**Fig. 5.**
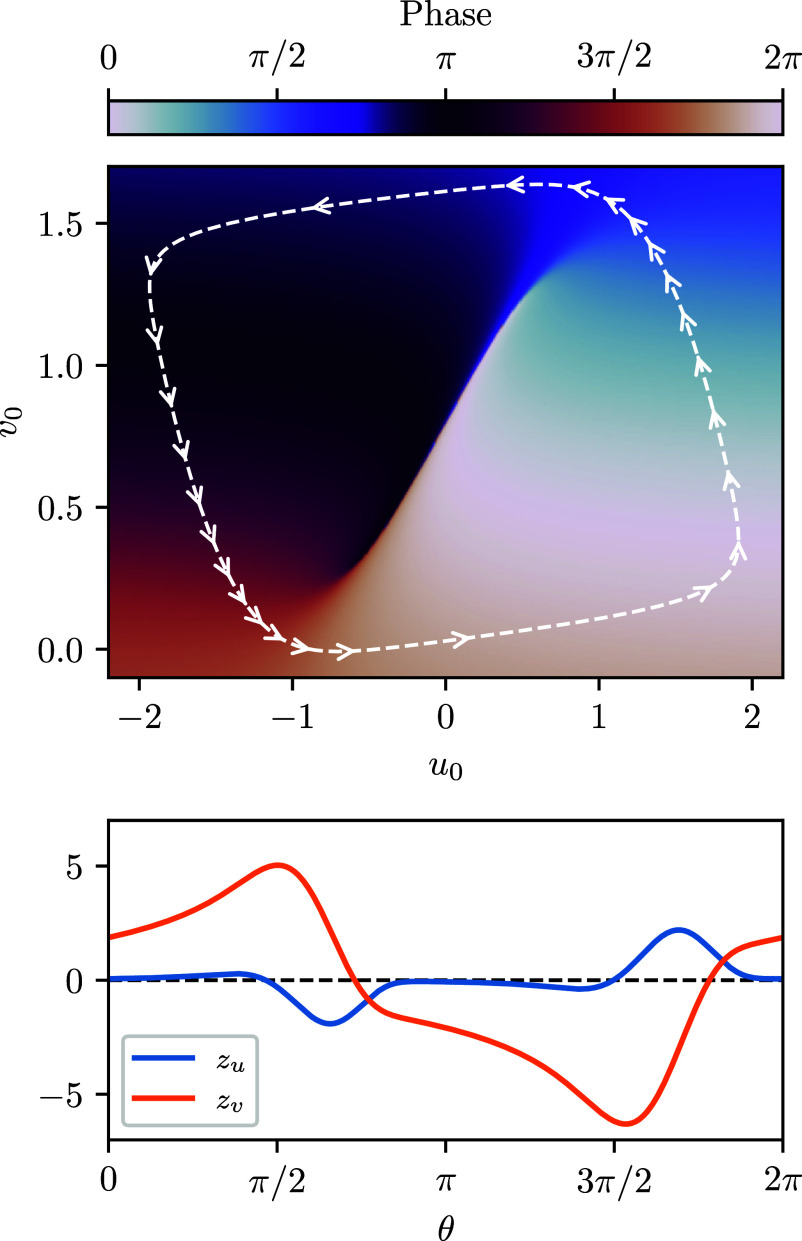
Phase sensitivity analysis of the FitzHugh-Nagumo model via discrete eigenvalue problem adjoints. *Top*: Limit cycle and explicitly computed phase function. Arrows on the limit cycle are equispaced in time, illustrating the slow-fast dynamics of the oscillator. *Bottom*: The phase sensitivity components measuring the gradient of the phase function along the limit cycle.

The limit cycle, the explicitly computed phase function, and the phase sensitivity as computed with our adjoint method are shown in Fig. 5 for parameters a=0.7, b=0.8, ϵ=0.08, and I=0.8 and demonstrate excellent agreement with ref. 87. This approach can be easily adapted to other equation sets, including PDEs, providing a systematic route to phase-reduction analysis for a wide range of oscillators.

## Discussion

We have presented a framework for producing fast adjoint solvers for sparse spectral methods and have demonstrated its implementation in the Dedalus library. Leveraging the structure of the underlying sparse algorithms, we have developed a hybrid approach in which adjoints are explicitly implemented for fundamental operations and composed via a high-level reverse-mode AD system. This enables the efficient evaluation of vector-Jacobian products for arbitrary nonlinear expressions based on their graph representations in Dedalus. By individually handling procedures such as linear system solves, spectral transforms, timestepping routines, eigenvalue solvers, and Newton solvers, we can quickly and reliably compute adjoints of PDE workflows that would otherwise be inefficiently traced by an AD compiler. This implementation is easily extendable by end users, who can add custom differentiable operators as simple Python classes.

Our implementation reuses data structures from the forward solver—such as matrix decompositions and transform plans—so that the resulting adjoint solver is both computationally and memory efficient. It fully supports all domains, problem types, high-order integrators, and parallelization strategies available in Dedalus, a distinct advantage over currently available differentiable solvers, e.g., JAX-CFD. This flexibility enables users to obtain adjoint information for general PDEs directly from their forward model implementations in Dedalus, without requiring additional libraries or restructuring of the original code.

To demonstrate the capabilities of this approach, we have reproduced canonical examples using adjoint methods from classical fluid mechanics, dynamo theory, modal analysis of turbulence, and mathematical neuroscience. These examples span a range of problem types and geometries, showcasing the versatility and advantages of a differentiable spectral framework. Specifically, our examples illustrate how such a code can be used for parametric sensitivity analysis, numerical continuation, nonlinear optimization, nonmodal stability theory, and phase sensitivity analysis. These methods are applicable across scientific disciplines and utilize gradient information in diverse ways. By extending open-source differentiable solvers to encompass modern spectral methods, this work enables adjoint-based studies in domains where spectral approaches are essential for accuracy and efficiency.

In this work, we have focused on first-order model derivatives and coupling to gradient-based optimizers. A natural next step is to automate the computation of higher-order derivatives for more general optimization routines, e.g., ref. 88. This would enable techniques such as Newton-type methods for optimization as well as uncertainty quantification and Bayesian inference, which can benefit from efficient Hessian-vector products for posterior sampling (see e.g., refs. 89 and 90). Additionally, it is now possible to combine Dedalus models with machine learning libraries by manually linking gradient computations. Creating a dedicated interface to simplify this integration—similar to the interface between Firedrake and PyTorch (91)—would enable seamless end-to-end model-based training for a wide range of applications.

Finally, an important future direction is the integration of adjoints with other ongoing extensions to Dedalus, including new bases for additional geometries and optimizations for graphics and tensor processing units. Key to this integration is the foundation we have established in rendering the code differentiable via high-level abstractions and modular design, rather than low-level implementations tied to a specific AD compiler. This design will allow adjoint capabilities to be maintained and extended efficiently across emerging platforms and optimization ecosystems.

## Materials and Methods

Solving the adjoint state equation, Eq. **5**, requires adjoint linear-system solves—e.g., for sparse matrices $A_{i}$—and the evaluation of vector-Jacobian products for the right-hand-side (RHS) terms J(X,p) and F(X,p). Here, we outline the approach used to efficiently automate these processes in Dedalus. For details on how different problem types map to this structure, see *SI Appendix*.

### Linear System Solves.

In each forward iteration, linear systems are solved using fast direct solvers, typically via sparse LU factorizations of $A_{i}$. The corresponding adjoint solves are performed by solving the adjoints of the LU factors of $A_{i}$, without requiring additional factorizations.

### Nonlinear Operator Graphs.

Nonlinear terms are represented in Dedalus as directed acyclic graphs (DAGs) of operators acting on the fields and parameters of the PDE. These graphs are evaluated via depth-first recursion, which triggers spectral transforms as needed to compute intermediate operators.

Forward-mode (tangent) sensitivity propagation through an operator graph is implemented as a matrix-free Jacobian-vector product (JVP) evaluated concurrently with the primal graph. This only requires each operator to provide its discrete derivative.

Reverse-mode (adjoint/cotangent) sensitivity propagation is implemented as a matrix-free vector-Jacobian product (VJP) utilizing the existing DAG operator structure. During the forward evaluation of the operator graph, a tape records the evaluation order of intermediate operators, forming a topological sort of the DAG. The VJP is computed by propagating the cotangents backward through this recorded evaluation order and accumulating at the root nodes. This approach is equivalent to reverse-mode automatic differentiation but is performed in a reliably memory- and compute-efficient manner by leveraging the high-level structure of the forward operator DAG.

**Fig. 6.**
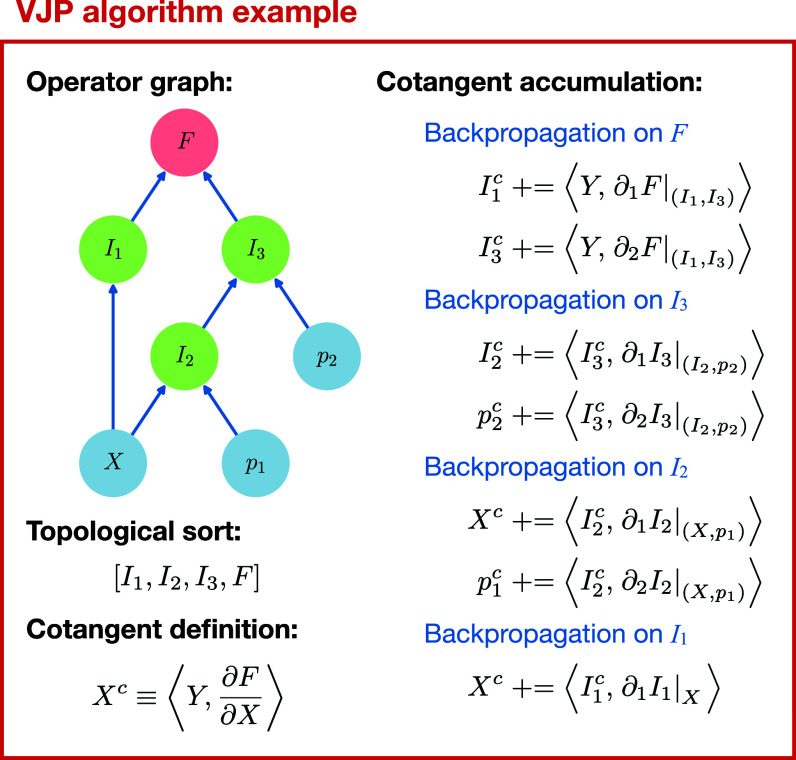
Example of evaluating a VJP via backpropagation through a symbolic operator graph. The graph is first forward-evaluated to compute the primal values of all intermediate operators $I_{j}$. This evaluation produces a topological sort of the graph. Cotangents for each intermediate result and each operand $\left(X,p_{j}\right)$ are accumulated by applying VJPs for each operation in the reverse order of the topological sort. Each VJP uses the cotangent of the respective operator and is evaluated using the primal values.

Fig. 6 illustrates this process for an explicit example graph structure. The backpropagation steps for linear operators are performed by directly applying the adjoints of the sparse operator matrices to the incoming cotangents. The backpropagation steps for local nonlinear operators, such as multiplication, are evaluated on a collocation grid using adjoint transforms, where the operator Jacobian is diagonal (separable over grid points). The cotangents for all variables are initialized to zero at the beginning of an adjoint step, and the contributions from VJPs of J and F are accumulated in-place to produce the full derivatives in Eq. **5**.

### Spectral Transforms.

During the evaluation of nonlinear operator graphs, spectral transforms are employed to efficiently apply differential operators to field coefficients in their sparse spectral representations, while nonlinearities are evaluated on a collocation grid. This pseudospectral approach is substantially faster than either pure-collocation or pure-spectral approaches when fast transforms are available.

When evaluating operator VJPs, the adjoint of each spectral transform must therefore be applied. To automate this process, we have implemented specialized “adjoint field” classes that represent cotangent quantities and overload transformation behaviors to automatically apply the appropriate adjoint transforms.

Let f denote a field’s values on the collocation grid and $\hat{f}$ its spectral coefficients. Let T denote the forward transform, such that $\hat{f}=Tf$ and $f=T^{-1}\hat{f}$. Denoting an adjoint field as $f^{†}$, we require that $\left\langle f^{†},f\right\rangle =\left\langle {\hat{f}}^{†},\hat{f}\right\rangle$. Using the above identities, we obtain the relationships

[20] $$\begin{array}{c} {\hat{f}}^{†}=T^{-†}f^{†},\;f^{†}=T^{†}{\hat{f}}^{†}, \end{array}$$

showing that the forward transform of an adjoint field is the adjoint of the original backward transform, and vice versa.

For transforms implemented directly as matrix multiplications, the adjoints are computed using the Hermitian transpose of the matrix. For fast transforms (e.g., FFTs), the adjoint is implemented using a corresponding fast transform, depending on the transform type.

### Supported Operators.

Many common operators are implemented in the Dedalus codebase, including differential operators, basis conversions, linear functionals for boundary conditions, and common nonlinear terms. The discrete JVPs and VJPs for linear operators generally use their matrix forms, and we have manually defined the JVP and VJP actions for each nonlinear operator. The correctness of these discrete derivatives are verified with unit tests.

Custom operators can be easily added by creating Python classes that inherit from the included operator base classes. For linear operators, only the operator matrix form needs to be specified. For nonlinear operators, the primal and differential actions are simply defined within methods on each custom class.

## Supplementary Material

Appendix 01 (PDF)

## Data Availability

Code for the example problems and instructions for installing all necessary software are available online (92).
