# Supplementary material for: Fast automated adjoints for spectral PDE solvers
Source: Proc Natl Acad Sci U S A. 2026 Apr 10;123(15):e2530440123. doi: 10.1073/pnas.2530440123 (PMC13080004; doi:10.1073/pnas.2530440123)
Supplement: Supplementary file 1 — Appendix 01 (PDF) [file pnas.2530440123.sapp.pdf]

Supporting Information for

Fast automated adjoints for spectral PDE solvers

Calum S. Skene[1] and Keaton J. Burns[2]

Correspondence should be addressed to both authors: [1]cskene3@ed.ac.uk, [2]kjburns@mit.edu.

Supporting Information Text

1. Problem types

Here we give further details of the adjoint approach used for each solver type, following the general PDE system formulations used in `Dedalus`.

A. Linear boundary value problems. `Dedalus` linear boundary value problems take the form

$$\mathbf{L}(\mathbf{p})\mathbf{X} = \mathbf{G}(\mathbf{p}), \tag{1}$$

or equivalently

$$\mathbf{F}(\mathbf{X},\mathbf{p}) \equiv \mathbf{L}(\mathbf{p})\mathbf{X} - \mathbf{G}(\mathbf{p}) = 0. \tag{2}$$

This system is solved by computing and solving the LU factorization of $\mathbf{L}$, which is generally sparse. The adjoint state equation is simply

$$\mathbf{L}(\mathbf{p})^\dagger \mathbf{Y} = \mathbf{H}, \tag{3}$$

for a right-hand side $\mathbf{H}$ that depends on the cost functional or upstream solves. The LU factorization of $\mathbf{L}$ computed for the forward solve can be reused for the adjoint solve. A VJP of $\mathbf{F}$ with cotangents $-\mathbf{Y}$ then propagates sensitivities to the parameters $\mathbf{p}$.

B. Nonlinear boundary value problems. `Dedalus` nonlinear boundary value problems take the form

$$\mathbf{L}(\mathbf{p})\mathbf{X} = \mathbf{G}(\mathbf{X},\mathbf{p}), \tag{4}$$

or equivalently

$$\mathbf{F}(\mathbf{X},\mathbf{p}) \equiv \mathbf{L}(\mathbf{p})\mathbf{X} - \mathbf{G}(\mathbf{X},\mathbf{p}) = 0. \tag{5}$$

This is solved via Newton-Kantorovich iterations using the Jacobian $\mathbf{J} = \partial_\mathbf{X}\mathbf{F}$. The adjoint state equation is obtained from the linearization of the final Newton step,

$$\underbrace{\left(\mathbf{L}(\mathbf{p}) - \frac{\partial \mathbf{G}}{\partial \mathbf{X}}(\mathbf{X},\mathbf{p})\right)^\dagger}_{\mathbf{J}(\mathbf{X},\mathbf{p})^\dagger} \mathbf{Y} = \mathbf{H}, \tag{6}$$

for a right-hand side $\mathbf{H}$ that depends on the cost functional or upstream solves. With direct solvers, the factorization of $\mathbf{J}$ from the final Newton iteration can be reused for the adjoint solve. With iterative solvers, JVPs and VJPs of $\mathbf{F}$ are used in the forward and adjoint solves, respectively. A VJP of $\mathbf{F}$ with cotangents $-\mathbf{Y}$ then propagates sensitivities to the parameters $\mathbf{p}$.

C. Eigenvalue problems. `Dedalus` eigenvalue problems take the generalized form

$$\left[\lambda \mathbf{M}(\mathbf{p}) + \mathbf{L}(\mathbf{p})\right]\mathbf{X} = 0, \tag{7}$$

$$\mathbf{X}^\dagger \mathbf{X} = 1, \tag{8}$$

and are solved using generalized eigenvalue routines given $\mathbf{M}$ and $\mathbf{L}$, or with shift-invert schemes based on LU factorizations of pencils $(\alpha\mathbf{M} + \mathbf{L})$. Treating this equation as a nonlinear equation in the augmented state $[\mathbf{X}, \lambda]$ for a simple eigenpair, the adjoint equation for the augmented adjoint state $[\mathbf{Y}, \sigma]$ is

$$\left[\bar{\lambda}\mathbf{M}^\dagger(\mathbf{p}) + \mathbf{L}^\dagger(\mathbf{p})\right]\mathbf{Y} + \sigma\mathbf{X} = \mathbf{H}_\mathbf{X}, \tag{9}$$

$$\mathbf{X}^\dagger \mathbf{M}^\dagger \mathbf{Y} = H_\lambda, \tag{10}$$

for right-hand sides $[\mathbf{H}_\mathbf{X}, H_\lambda]$ that depend on the cost functional or upstream solves.

The sensitivity of the eigenvalue λ is determined by considering the cost functional $J = \lambda$, which yields $[\mathbf{H}_\mathbf{X}, H_\lambda] = [0, 1]$. By the Fredholm alternative, the adjoint state equation is only solvable when $\sigma = 0$, and the solution $\mathbf{Y}$ is the left eigenvector associated with $\mathbf{X}$. Left and right eigenvectors can both be supplied by the underlying sparse and dense eigenvalue routines

wrapped in `Dedalus`. A VJP of Eq. (7) with cotangents $-[\mathbf{Y}, \sigma]$ then propagates sensitivities to the parameters $\mathbf{p}$. We note this procedure automatically implements the classical eigenvalue perturbation equation (see (1), for example):

$$\frac{\mathrm{d}\lambda}{\mathrm{d}\mathbf{p}} = -\left\langle \mathbf{Y}, \left(\lambda \frac{\partial \mathbf{M}}{\partial \mathbf{p}} + \frac{\partial \mathbf{L}}{\partial \mathbf{p}}\right) \mathbf{X} \right\rangle. \quad [11]$$

By the implicit function theorem, the eigenvector sensitivity can then be computed by solving

$$[\lambda \mathbf{M}(\mathbf{p}) + \mathbf{L}(\mathbf{p})] \frac{\mathrm{d}\mathbf{X}}{\mathrm{d}\mathbf{p}} = -\left(\mathbf{M}\frac{\mathrm{d}\lambda}{\mathrm{d}\mathbf{p}} + \lambda \frac{\mathrm{d}\mathbf{M}}{\mathrm{d}\mathbf{p}} + \frac{\mathrm{d}\mathbf{L}}{\mathrm{d}\mathbf{p}}\right) \mathbf{X}. \quad [12]$$

This is a singular equation when λ is a generalized eigenvalue, but is solvable with a unique eigenvector sensitivity that is orthogonal to $\mathbf{X}$. This sensitivity analysis naturally carries forward to higher order derivatives, as outlined by (2).

D. Initial value problems. `Dedalus` initial value problems take the form

$$\mathbf{M}(\mathbf{p})\partial_t \mathbf{X} + \mathbf{L}(\mathbf{p})\mathbf{X} = \mathbf{F}(\mathbf{X}, \mathbf{p}, t), \quad [13]$$

which are either integrated via a multistep IMEX scheme (3) or a Runge-Kutta IMEX scheme (4). For both of these timestepping classes, each step requires computing the explicit terms $\mathbf{F}$, solving linear systems, and updating the state $\mathbf{X}$. To provide the adjoint of this sequence, we have manually implemented the discrete adjoints of the timestepping base classes (for an example of how to derive the adjoint timestepping schemes, see (5)).

An s-step multistep IMEX scheme takes the form

$$(a_0^n \mathbf{M} + b_0^n \mathbf{L})\mathbf{X}_n = \sum_{i=1}^{s} \left[c_i^n \mathbf{F}(\mathbf{X}_{n-i}) - (a_i^n \mathbf{M} + b_i^n \mathbf{L})\mathbf{X}_{n-i}\right], \quad [14]$$

where the coefficients (a_i^n, b_i^n, c_i^n) depend on the timestep at step n and revert to lower-order multistep IMEX schemes for early iterations without sufficient history. The adjoint of this scheme is

$$(a_0^n \mathbf{M} + b_0^n \mathbf{L})^\dagger \mathbf{Y}_n = \sum_{i=1}^{s} \left(c_i^{n+i} \frac{\partial \mathbf{F}(\mathbf{X}_n)}{\partial \mathbf{X}_n} - (a_i^{n+i}\mathbf{M} + b_i^{n+i}\mathbf{L})\right)^\dagger \mathbf{Y}_{n+i}. \quad [15]$$

Similarly, stiffly accurate Runge-Kutta IMEX schemes with s stages can be written as

$$(\mathbf{M} + \mathrm{d}t^n H_{i,i}\mathbf{L})\mathbf{X}_{n,i} = \mathbf{M}\mathbf{X}_{n,0} + \mathrm{d}t^n \sum_{j=0}^{i-1} [A_{i,j}\mathbf{F}_{n,j} - H_{i,j}\mathbf{L}\mathbf{X}_{n,j}], \quad [16]$$

with $\mathbf{X}_n = \mathbf{X}_{n,0} = \mathbf{X}_{n-1,s}$ and where $\mathrm{d}t^n$ is the timestep at iteration n. This has the adjoint

$$(\mathbf{M} + \mathrm{d}t^n H_{i,i}\mathbf{L})^\dagger \mathbf{Y}_{n,i} = \begin{cases} \mathbf{Y}_{n+1,0}, & \text{if } i = s, \\ \sum_{j=i+1}^{s} \mathrm{d}t^n \left(A_{j,i}\frac{\partial \mathbf{F}_{n,i}}{\partial \mathbf{X}_{n,i}} - H_{j,i}\mathbf{L}\right)^\dagger \mathbf{Y}_{n,j}, & \text{if } 0 < i < s, \end{cases} \quad [17]$$

with

$$\mathbf{Y}_{n,0} = \sum_{j=1}^{s} \left(\mathbf{M}^\dagger + \mathrm{d}t^n \left[A_{j,0}\frac{\partial \mathbf{F}_{n,0}}{\partial \mathbf{X}_{n,0}} - H_{j,0}\mathbf{L}\right]^\dagger\right) \mathbf{Y}_{n,j}, \quad [18]$$

and where $\mathbf{Y}_n = \mathbf{Y}_{n,0} = \mathbf{Y}_{n-1,s}$.

In both cases we see that an adjoint step requires computing VJPs of $\mathbf{F}$ with given cotangents, solving the adjoints of the linear systems from the forward evolution, and updating the cotangents. The total adjoint integration is a sequence of adjoint steps with the same timestepping history as in the forward integration. The LU factorizations from the forward integration are reused where possible, i.e., unless they must be rebuilt due to a change in the timestep. The VJPs of $\mathbf{F}$ are performed using the automatic differentiation technique discussed in the manuscript. As the values of the state variables at each iteration of the forward integration are required to compute VJPs in the adjoint integration, checkpointing can be employed via the `checkpoint_schedules` library (6), which includes many state-of-the-art checkpointing schemes (7–12). This allows the user to easily choose between memory and disk based checkpointing.

The adjoint timestepping schemes propagate sensitivities backwards in time from the cotangents defined at the final time. These final-time cotangents come from the cost functional or other upstream calculations such as LBVPs, NLVPs or other IVPs. The sensitivities with respect to parameters $\mathbf{p}$ are accumulated during the adjoint integration via VJPs of $\mathbf{F}$, $\mathbf{MX}$, and $\mathbf{LX}$ with $\mathbf{Y}_n$ and $\mathbf{Y}_{n,i}$, as appearing in Eq. (14) and Eq. (16), respectively. If the cost functional depends on a quantity $\mathcal{I}$ integrated over time, we can compute this by adding the ODE $\partial_t \mathcal{C} = \mathcal{I}$ to the PDE system. The integrated quantity $\mathcal{C}(T)$ is then obtained with the same accuracy as the forward temporal integration, and its influence on the cotangent calculation is naturally included in the adjoint timestepping routine via VJPs applied to $\mathcal{I}$.

2. Example implementation details

Here we give further details of the implementations for each example.

A. Parametric sensitivity and numerical continuation. The plane-Poiseuille problem is discretized in the wall-normal direction with 256 Chebyshev polynomials. Streamwise variations are parameterized by the wavenumber α. A sparse eigenvalue solve is performed with the `scipy.sparse.eigs` routine which wraps `ARPACK`.

B. Nonlinear optimization. In the optimal dynamo problem, the ball is discretized with spin-weighted spherical harmonics in longitude and latitude and one-sided Jacobi polynomials in radius (13). We use spherical harmonics up to degree $\ell = 15$ and radial modes up to degree 31. Nonlinear terms are computed pseudospectrally using a 3/2-dealiasing rule. A second-order multistep IMEX scheme (SBDF2) is used with fixed timestep $\Delta t = 5 \times 10^{-4}$. We have checked that increasing the resolution and decreasing the timestep does not significantly affect the results. The direct solution is provided by solving an LBVP to obtain $\mathbf{u}$ from $\boldsymbol{\omega}$ and then integrating an IVP to evolve either $\mathbf{A}$ or $\mathbf{B}$. The adjoint solve requires propagating cotangents back from the cost functional through the IVP and then through the LBVP. This example can be run with either memory or disk-based checkpointing using the "H-revolve" scheduling algorithm (11).

C. Resolvent analysis. In the pipe-flow problem, the radial direction in the disk is discretized with Zernike polynomials (14) up to a maximum degree of 127. The azimuthal and streamwise variations are parameterized by wavenumbers m and k. A spectrally accurate weight matrix $\mathbf{W}$ for the L^2-norm is generated with Zernike quadrature. The action of the resolvent matrix $\boldsymbol{\mathcal{H}}$ on a forcing vector $\hat{\mathbf{f}}$ is obtained by solving an LBVP. The optimal forcing that maximizes the Rayleigh quotient

$$\sigma = \frac{\|\boldsymbol{\mathcal{H}}\hat{\mathbf{f}}\|_{\mathbf{W}}}{\|\hat{\mathbf{f}}\|_{\mathbf{W}}} \qquad [19]$$

is found by computing the singular value decomposition (SVD) of $\boldsymbol{\mathcal{H}}_{\mathbf{M}} = \mathbf{M}^{-1}\boldsymbol{\mathcal{H}}\mathbf{M}$, where $\mathbf{M}$ is the Cholesky decomposition of the weight matrix, $\mathbf{W} = \mathbf{M}^{\dagger}\mathbf{M}$ (trivial here since $\mathbf{W}$ is diagonal). Finding the SVD requires applications of both $\boldsymbol{\mathcal{H}}_{\mathbf{M}}$ and $\boldsymbol{\mathcal{H}}_{\mathbf{M}}^{\dagger}$, the latter of which uses the adjoint solver for the LBVP for $\boldsymbol{\mathcal{H}}$.

D. Phase reduction analysis. In the FitzHugh-Nagumo problem, the full phase function was computed by integrating the system over a grid of initial conditions. For the phase sensitivity analysis, time is discretized with 512 Fourier modes. The nonlinear terms are computed on a grid of size 1024 to dealias the cubic nonlinearities.

3. Verification

To verify the correctness of our adjoint implementations, we have performed several tests of the accuracy of our computed model gradients.

For the plane-Poiseuille and optimal dynamo examples, we use the Taylor-remainder test (see (15), for example) based on the expansion of the target functional

$$\mathcal{J}(\mathbf{p} + \epsilon\mathbf{p}') = \mathcal{J}(\mathbf{p}) + \epsilon\langle \mathbf{Y}, \mathbf{p}' \rangle + \mathcal{O}(\epsilon^2), \qquad [20]$$

where $\mathbf{p}'$ is a random parameter perturbation and $\mathbf{Y}$ is the numerically computed model sensitivity. This test confirms the correctness of $\mathbf{Y}$ by checking that

$$|\mathcal{J}(\mathbf{p} + \epsilon\mathbf{p}') - \mathcal{J}(\mathbf{p}) - \epsilon\langle \mathbf{Y}, \mathbf{p}' \rangle| = \mathcal{O}(\epsilon^2). \qquad [21]$$

This provides a simple and effective test for verifying cotangent calculations and can be applied to linear and nonlinear problems. We plot these Taylor remainders for a range of values of ϵ in Fig. S1, and list the best-fit power law exponents in Table S1. We see that the remainders decay at second order over a wide range of perturbation amplitudes, verifying the accuracy of the discrete adjoints.

For the other examples, direct error metrics are shown in Table S2, and again indicate that the discrete adjoints are computed correctly. For the pipe-flow example, we use the fact that $\boldsymbol{\mathcal{H}}_{\mathbf{M}}$ is a linear operator to directly verify the adjoint relation

$$\langle \mathbf{Y}, \boldsymbol{\mathcal{H}}_{\mathbf{M}}\mathbf{X} \rangle = \langle \boldsymbol{\mathcal{H}}_{\mathbf{M}}^{\dagger}\mathbf{Y}, \mathbf{X} \rangle. \qquad [22]$$

For the FitzHugh-Nagumo equation, we check that the tangential phase tendency is one at all times:

$$z_u \frac{\mathrm{d}u_0}{\mathrm{d}t} + z_v \frac{\mathrm{d}v_0}{\mathrm{d}t} = 1. \qquad [23]$$

This test requires that the phase sensitivity function is precisely the gradient of the phase function on the limit cycle (16).

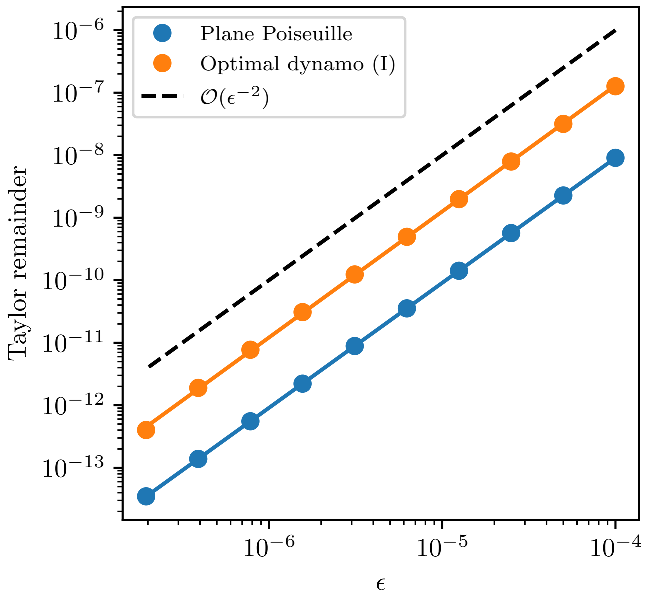

Fig. S1. Taylor remainders demonstrating the convergence of finite difference estimates of the model gradient towards our adjoint results. Second-order convergence (dashed line) indicates proper implementation of the discrete adjoints.

Problem	Taylor remainder exponents
Plane Poiseuille	1.999
Optimal dynamo (I)	2.016
Optimal dynamo (II)	1.993

Table S1. Best-fit Taylor-remainder exponents demonstrating the correctness of the discrete adjoint implementations. Two setups were tested for the optimal dynamo problem. Setup I: a second-order multistep IMEX scheme without checkpointing. Setup II: a second-order Runge-Kutta IMEX scheme using the "H-revolve" checkpointing schedule with 400 checkpoints in RAM and 50 checkpoints on disk.

Problem	Error
Pipe flow	1.05×10^{-14}
FitzHugh-Nagumo	2.15×10^{-13}

Table S2. Direct error measurements for several example problems. For the pipe-flow problem, we report the inner-product test error. For the FitzHugh-Nagumo problem, we report the L^∞ error over time of the tangential phase tendency.

References

1. P Luchini, A Bottaro, Adjoint equations in stability analysis. *Annu. Rev. Fluid Mech.* **46**, 493–517 (2014).
2. GA Mensah, A Orchini, JP Moeck, Perturbation theory of nonlinear, non-self-adjoint eigenvalue problems: Simple eigenvalues. *J. Sound Vib.* **473**, 115200 (2020).
3. D Wang, SJ Ruuth, Variable step-size implicit-explicit linear multistep methods for time-dependent partial differential equations. *J. Comput. Math.* **26**, 838–855 (2008).
4. UM Ascher, SJ Ruuth, RJ Spiteri, Implicit-explicit Runge-Kutta methods for time-dependent partial differential equations. *Appl. Numer. Math.* **25**, 151–167 (1997) Special Issue on Time Integration.
5. PM Mannix, CS Skene, D Auroux, F Marcotte, A robust, discrete-gradient descent procedure for optimisation with time-dependent PDE and norm constraints. *The SMAI J. Comput. Math.* **10**, 1–28 (2024).
6. DI Dolci, JR Maddison, DA Ham, G Pallez, J Herrmann, checkpoint_schedules: schedules for incremental checkpointing of adjoint simulations. *J. Open Source Softw.* **9**, 6148 (2024).
7. P Stumm, A Walther, Multistage approaches for optimal offline checkpointing. *SIAM J. on Sci. Comput.* **31**, 1946–1967 (2009).
8. G Aupy, J Herrmann, P Hovland, Y Robert, Optimal multistage algorithm for adjoint computation. *SIAM J. on Sci. Comput.* **38**, C232–C255 (2016).
9. G Aupy, J Herrmann, Periodicity in optimal hierarchical checkpointing schemes for adjoint computations. *Optim. Methods Softw.* **32**, 594–624 (2017).
10. G Pringle, D Jones, S Goswami, S Narayanan, D Goldberg, Providing the ARCHER community with adjoint modelling tools for high-performance oceanographic and cryospheric computation, Technical report (2016).
11. J Herrmann, GP Aupy, H-Revolve: A framework for adjoint computation on synchronous hierarchical platforms. *ACM Trans. Math. Softw.* **46** (2020).
12. JR Maddison, Step-based checkpointing with high-level algorithmic differentiation. *J. Comput. Sci.* **82**, 102405 (2024).
13. GM Vasil, D Lecoanet, KJ Burns, JS Oishi, BP Brown, Tensor calculus in spherical coordinates using Jacobi polynomials. part-I: Mathematical analysis and derivations. *J. Comput. Physics: X* **3**, 100013 (2019).
14. GM Vasil, et al., Tensor calculus in polar coordinates using Jacobi polynomials. *J. Comput. Phys.* **325**, 53–73 (2016).
15. PE Farrell, DA Ham, SW Funke, ME Rognes, Automated derivation of the adjoint of high-level transient finite element programs. *SIAM J. on Sci. Comput.* **35**, C369–C393 (2013).
16. GB Ermentrout, D Terman, *Mathematical Foundations of Neuroscience*, Interdisciplinary Applied Mathematics. (Springer, New York, NY) Vol. 35, (2010).
